# Additive interaction between potentially modifiable risk factors and ethnicity among individuals in the Han, Tujia and Miao populations with first-ever ischaemic stroke

**DOI:** 10.1186/s12889-021-11115-x

**Published:** 2021-06-03

**Authors:** Na Zhang, Xinrui Wu, Mengyuan Tian, Xiaolei Wang, Jian Ding, Yong Tian, Chengcai Liang, Zhi Zeng, Hua Xiang, Hongzhuan Tan

**Affiliations:** 1 Hunan Provincial Key Laboratory of Clinical Epidemiology, Xiangya School of Public Health, Central South University, Changsha, Hunan China; 2 Department of Epidemiology and Health Statistics, Xiangya School of Public Health, Central South University, Changsha, Hunan China; 3 Hunan Provincial Institute of Geriatrics, Hunan Provincial People’s Hospital (the First Affiliated Hospital of Hunan Normal University), Changsha, Hunan China; 4 Department of Neurology, the First Affiliated Hospital of Jishou University, the Tujia-Miao autonomous prefecture of Xiangxi, Hunan China; 5 Interventional Radiology Center, Hunan Provincial People’s Hospital (the First Affiliated Hospital of Hunan Normal University), Changsha, Hunan China

**Keywords:** Ischaemic stroke, Risk factors, Primary prevention, Ethnicity-specific, Interaction, Epidemiology

## Abstract

**Background:**

As a country with one-fifth of the global population, China has experienced explosive growth in ischaemic stroke (IS) burden with significant ethnic and geographic disparities. The aim of this study was to examine the differences in potentially modifiable risk factors for ischaemic stroke among the Han population and two ethnic minorities (Tujia and Miao).

**Methods:**

A case-control study was conducted with 324 cases of first-ever ischaemic stroke from the hospitals of the Xiangxi Tujia and Miao Autonomous Prefecture and 394 controls from communities covering the same area between May 1, 2018, and April 30, 2019. Structured questionnaires were administered, and physical examinations were performed in the same manner for cases and controls. Univariate and multivariate logistic regression analyses with adjusted odds ratios (ORs) and 95% confidence intervals (CIs) were used to examine the association between risk factors and ischaemic stroke. An additive model was used to study the interaction between the modifiable risk factors and ethnicity with R software.

**Results:**

Higher high-sensitivity C-reactive protein levels (OR 50.54, 95%CI 29.76–85.85), higher monthly family income (4.18, 2.40–7.28), increased frequency of hot pot consumption (2.90, 1.21–6.93), diabetes mellitus (2.62, 1.48–4.62), a higher apolipoprotein (Apo)B/ApoA1 ratio (2.60, 1.39–4.85), hypertension (2.52, 1.45–4.40) and moderate-intensity physical activity (0.50, 0.28–0.89) were associated with ischaemic stroke. There was an additive interaction between the ApoB/ApoA1 ratio and ethnicity in the Tujia and Miao populations with first-ever ischaemic stroke (the relative excess risk due to the interaction was 5.75, 95% CI 0.58 ~ 10.92; the attributable proportion due to the interaction was 0.65, 95% CI 0.38 ~ 0.91; the synergy index was 3.66, 95% CI 1.35 ~ 9.93).

**Conclusions:**

This is the first case-control study examining modifiable risk factors for ischaemic stroke among the Han population and two ethnic minorities (Tujia and Miao) in China. Some differences were observed in the impact of risk factors among these ethnic groups. Our results may help interpret health-related data, including surveillance and research, when developing strategies for stroke prevention.

**Supplementary Information:**

The online version contains supplementary material available at 10.1186/s12889-021-11115-x.

## Background

Ischaemic stroke (IS) occurs when a vessel supplying blood to the brain is obstructed, and IS accounts for 69.6 and 77.8% of the total stroke incidence and prevalence in China, respectively [1]. With the acceleration of social ageing and urbanization, the popularity of unhealthy lifestyles among residents, and the widespread exposure to cerebrovascular risk factors, the stroke burden in China has shown explosive growth over the past 30 years. IS is characterized by rapid growth in low-income populations, distinct sex- and region-related differences, and a younger trend of incidence [1, 2]. Ten modifiable risk factors for IS, in descending order of importance, are hypertension (HT), the apolipoprotein (Apo)B/ApoA1 ratio, physical activity, diet, the waist-to-hip ratio (WHR), psychosocial factors, smoking, cardiac causes, diabetes mellitus (DM), and alcohol consumption, all of which accounted for 91.5 and 95.2% of the population attributable risk (PAR) of IS worldwide and in China, respectively, as quantified by the INTERSTROKE investigators [3]. In this regard, it is suggested that a large proportion of the IS burden may have been preventable through control of the above 10 traditional modifiable risk factors with population-level primary interventions for decades. However, the Global Burden of Disease 2017 Study (GBD2017) reported that stroke remains the leading cause of death and disability-adjusted life-years at the national level in China and the third leading cause of global years of life lost [4, 5].

According to the Framingham Heart Study, stroke incidence in the US has declined over time; however, the cohort was predominantly Caucasian [6], and the racial and geographic disparities were significant, with African American and Hispanic populations experiencing the greatest disease burden [7]. There is also a north-to-south gradient in stroke burden in China, with the greatest stroke burden observed in the northern and central regions [1, 8]. Some differences in the impact of risk factors for IS were demonstrated between black Caribbean and African populations in a European case-control study [9]. The INTERSTROKE investigators also found regional and ethnic variations in the relative importance of most individual risk factors for IS, which may contribute to global variations in the frequency of IS and thus support the development of population-specific measures to prevent IS [3]. Although the reasons for ethnic differences in stroke incidence and mortality are not entirely clear, the role of the distribution of risk factor burden across ethnic groups must be considered an important contributor. China is a unified multi-ethnic country with 56 ethnic groups, of which the Han is the largest group, and the other 55 ethnic groups are referred to as ethnic minorities. Data on IS in ethnic minorities are sparse in China, and characteristics such as risk factor profiles from one ethnic group may not be representative of those in another. Many of the Chinese ethnic minorities are quite similar to the Han in language and culture, but others are quite different both from the Han and from one another in some very important features, such as diet, attire, marriage, customs and lifestyle. Perhaps ethnicity, which is not proven to be a surrogate for genetic constitution, may interact with traditional risk factors for IS. Additive interaction has been shown to be relevant for evaluating prevention or intervention strategies in public health decision-making [10, 11].

According to the sixth national population census in 2010, the Miao (9.43 million) and Tujia (8.35 million) minorities were ranked as the fifth and seventh largest minority groups, respectively, but few reliable data are available to identify the differences in risk factors for IS among the Tujia, Miao, and Han populations. This study aimed to investigate the differences in the risk factors for IS among the Tujia, Miao, and Han populations and their additive interactions with ethnicity in the Xiangxi Tujia and Miao Autonomous Prefecture and to provide new insights into the risk factors, prevention and management strategies for IS among the Tujia, Miao, and Han ethnicities.

## Methods

### Study design and setting

A case-control design was utilized for this study. The study was carried out in Xiangxi Tujia and Miao Autonomous Prefecture, located in western Hunan Province and adjacent to Hubei and Guizhou Provinces, where people of Tujia and Miao constitute 80% of the total population of 2.98 million. We calculated the sample size for an unmatched case-control study based on the assumption as follows: α = 0.05, β = 0.2, OR = 2.0 (the minimum odds ratio (OR) of DM, which is one of the most important risk factors for IS), and a two-sided test. The exposure rate of DM in the control group was estimated as 15% based on other epidemiological studies in China, and the required minimum sample was found to be 277. Therefore, 324 IS patients and 394 controls were recruited to ensure logistic regression model validity, since some statisticians recommend an even more stringent “rule of thumb” of 20 samples per independent variable [12], and 15 risk factors for interest were collected in the study.

### Participants and recruitment

Participants were recruited between May 1, 2018, and April 30, 2019, from all 8 counties of the Xiangxi Tujia and Miao Autonomous Prefecture (Jishou, Baojing, Fenghuang, Guzhang, Huayuan, Longshan, Luxi, and Yongshun). All participants were residents who had lived in Xiangxi for more than 5 years. The case group included all IS patients who were admitted to the First Affiliated Hospital of Jishou University and all 8 County People’s Hospitals of the Xiangxi Tujia and Miao Autonomous Prefecture within 5 days of symptom onset, a 72 h hospital stay and a discharge diagnosis of first-ever IS. IS patients were defined as having “rapidly developing clinical signs of focal (at times global) disturbance of cerebral function, lasting more than 24 h or leading to death with no apparent cause other than that of vascular origin” according to the World Health Organization (WHO) clinical criteria [13]. Computed tomography (CT) or magnetic resonance imaging (MRI) of the brain was completed within 1 week of presentation. The exclusion criteria included those who (1) were unable to communicate and did not have a valid surrogate respondent; (2) had subdural haemorrhage, tumour, or brain abscess (i.e., non-vascular causes); (3) were under current hospitalization for acute coronary syndrome or myocardial infarction; and (4) were unable to provide consent or allow a surrogate to provide consent. For patients unable to communicate adequately, proxy respondents were used. We defined a valid proxy respondent as a spouse or first-degree relative who was living in the same home or was aware of the participant’s previous medical history and current treatments.

Controls were community-based from all 8 counties of Xiangxi and had no history of stroke. The age distribution of the controls was the same as that of IS patients. There was at least 1 control for every recruited case. The exclusion criteria for controls were identical to those described for cases.

The study was approved by the ethics committees of the Xiangya School of Public Health Central South University (XYGW-2018-26). All participants, or their proxy respondent, provided written informed consent before participating in the study.

### Data collection

According to the Trial of Org 10,172 in Acute Stroke Treatment (TOAST) criteria [14], IS was divided into 1) large-artery atherosclerosis, 2) cardioembolism, 3) small-vessel occlusion, 4) stroke of other determined aetiology, and 5) stroke of undetermined aetiology. Disease severity was estimated by the National Institutes of Health Stroke Scale (NIHSS) score [15] on admission by the physician. To standardize the assessment of stroke subtypes, all patients were adjudicated by a stroke specialist. All patients also underwent a range of blood tests, chest radiography, and electrocardiography at admission. Data on occupation, education, fertility, monthly family income, smoking status, frequency of fast food consumption, frequency of hot pot consumption, moderate-intensity physical activity (MIPA), and history of HT, DM and hyperlipidaemia were collected by the study physicians using structured questionnaires (Supplementary File 1) in IS patients and controls. Occupation was classified as either mental or manual work. Education was classified as either < 9 years of school education or ≥ 9 years (senior high school or above). The history of fertility was classified as having given birth to ≤2 children or to ≥3 children. Smoking status was classified as never smoked and current smoking (smoking ≥1 cigarette per day within the year prior to the interview, including those who had quit smoking less than a year prior). MIPA was defined as 4 h or more per week, including brisk walking, dancing, gardening, housework and domestic chores, traditional hunting and gathering, general building tasks (e.g., roofing, thatching, painting), carrying/moving moderate loads (< 20 kg), and so on [16]. HT was defined as under treatment with antihypertensive medication, a previous HT diagnosis, or current HT according to the 2003 WHO criteria (blood pressure of 140/90 mmHg or higher). For those with a history of DM or treated diabetes preceding IS, diabetes was defined according to the 1999 WHO criteria as fasting plasma glucose (FPG) level ≥ 7.0 mmol/L (126 mg/dL), a 2-h oral glucose tolerance test of ≥11.1 mmol/L (200 mg/dL), or glycated haemoglobin (HbA1c) level ≥ 6.5%. We defined dichotomous components of hyperlipidaemia as total cholesterol (TC) level ≥ 6.2 mmol/L (240 mg/dL), triglyceride (TG) level ≥ 2.3 mmol/L (200 mg/dL), low-density lipoprotein cholesterol (LDL-C) level ≥ 4.1 mmol/L (160 mg/dL), or high-density lipoprotein cholesterol (HDL-C) level < 1.0 mmol/L (40 mg/dL) [17].

Waist and hip circumferences were measured in the standing and supine positions. If patients were unable to stand because of disability, these measurements were completed only in the supine position [18]. Fasting blood samples (10 mL) were taken from cases and controls within 72 h of recruitment, separated by centrifugation, and frozen at − 70 °C immediately after processing. Samples were shipped in packaging incorporating dry ice cooling agents by courier from every site to a blood storage site, where they were stored at − 70 °C. FPG, HbA1c, TC, TG, LDL-C, HDL-C, ApoA1, ApoB and high-sensitivity C-reactive protein (hs-CRP) concentrations were measured at Dian Diagnostics Group with a Beckman Coulter (Brea, CA) AU680 Clinical Chemistry Analyzer and Beckman Coulter reagents. Detailed cut-offs for the WHR [19], HDL-C level [17], ApoB/ApoA1 ratio [20] and hs-CRP level [21] appear in Supplementary File 2: Table S1.

This study focused on whether the effects of traditional risk factors for IS are different among different ethnic groups, which will be of great importance for the development of population-specific measures to prevent IS among different ethnic groups. This study mainly compares Tujia, Miao and Han with each other.

### Statistical analysis

Statistical analyses were performed with IBM SPSS Statistics for Windows (version 23.0; Armonk, NY) and R for Windows (Version 4.0.3). All statistical tests were two-sided.

First, we performed a comparison between case and control groups using chi-squared tests and calculated univariate ORs and 95% confidence intervals (CIs) for all dichotomized or multinomial risk factors. Second, binary multivariable logistic regression was applied, with backward stepwise (likelihood ratio) variable removal at the level of *P* < 0.10, and adjusted ORs and 95% CIs were calculated. The Breslow-Day test was used for the homogeneity of the association between modifiable risk factors and IS by ethnicity [22]. If *P* < 0.05, we detected the biological interaction between the modifiable risk factor and ethnicity. Three measures of biological interaction—the relative excess risk due to interaction (RERI), the attributable proportion due to interaction (AP), and the synergy index (SI) [23]—with corresponding 95% CIs were calculated in R [24]. A RERI and AP equal to 0 and SI equal to 1 were defined as no interaction [23]. The names and values of variables in univariate and multivariate logistic regression analyses also appear in Supplementary File 2: Table S1.

## Results

### Basic information

The study included 324 patients and 394 controls, with ages ranging from 22 to 80 years. Descriptive characteristics of the patients, including age, sex, TOAST classification, and NIHSS score, are reported in Table 1. One hundred seventy (50.1%) patients had minor stroke, and 115 (33.9%) had moderate stroke.

**Table 1:** Demographic and Clinical Characteristics of Cases by Ethnic Group

	All(*N* = 324)	Tujia(*N* = 178)	Miao(*N* = 95)	Han(*N* = 51)
Age (years)	61.6 (11.4)	62.4 (11.2)	60.9 (12.3)	60.3 (10.2)
Women	98 (30.2)	62 (34.8)	26 (27.4)	10 (19.6)
TOAST classification				
Large-artery atherosclerosis	134 (41.4)	71 (39.9)	41 (43.2)	22 (43.1)
Cardioembolism	15(4.6)	13(7.3)	2(2.1)	–
Small-vessel occlusion	168 (51.9)	90 (50.6)	50 (52.6)	28 (54.9)
Stroke of other determined etiology	4(1.2)	2(1.1)	1(1.1)	1(2.0)
Stroke of undetermined etiology	3(0.9)	2(1.1)	1(1.1)	–
NIHSS score				
0(no stroke symptoms)	45 (13.3)	26 (14.6)	10 (10.5)	6 (11.8)
1–4(minor stroke)	170 (50.1)	81 (45.5)	58 (61.1)	25 (49.0)
5–15(moderate stroke)	115 (33.9)	66 (37.1)	27 (28.4)	18 (35.3)
16–20(moderate to severe stroke)	9(2.7)	5(2.8)	–	2(3.9)
CT or MRI of brain	324 (100.0)	178 (100.0)	95 (100.0)	51 (100.0)
ECG	324 (100.0)	178 (100.0)	95 (100.0)	51 (100.0)

Date are mean (SD) or number(%). *Abbreviation*: TOAST indicates Trial of Org 10,172 in Acute Stroke Treatment, *NIHSS* National Institutes of Health Stroke Scale, *CT* Computed tomography, *MRI* magnetic resonance imaging, *ECG* electrocardiography

### Univariate analysis of risk factors for first-ever ischaemic stroke

Table 2 provides the univariate analysis results for the entire population. We found all 15 risk factors to be significantly associated with first-ever IS. A higher hs-CRP level, a higher ApoB/ApoA1 ratio, DM, a higher monthly family income, HT, an increased frequency of fast food consumption, a lower HDL-C level, an increased frequency of hot pot consumption, hyperlipidaemia, a higher education level, a higher WHR, current smoking, and increased fertility among women were all associated with an increased risk of IS, and both manual work and moderate-intensity physical activity were associated with a reduced risk of IS.

**Table 2:** Univariate Analysis of Risk Factors for First-Ever Ischaemic Stroke in entire population

Risk factor	Case(N = 324) n (%)	Control(*N* = 394) n (%)	Crude OR(95%CI)
Manual Worker	257 (79.3%)	342 (86.8%)	0.58 (0.39–0.87)
Education ≥9 years	98 (30.2%)	86 (21.8%)	1.55 (1.11–2.18)
Given birth to 3 or more children	160 (49.4%)	163 (41.4%)	1.38 (1.03–1.86)
Monthly family income≥¥5000	148 (45.7%)	71 (18.0%)	3.83 (2.73–5.36)
Current smoking	155 (47.8%)	155 (39.3%)	1.41 (1.05–1.90)
Eating fast food frequency ≥ once per week	41 (12.7%)	16(4.1%)	3.42 (1.88–6.22)
Eating hot pot frequency ≥ once per week	40 (12.3%)	24(6.1%)	2.17 (1.28–3.69)
MIPA	228 (70.4%)	318 (80.7%)	0.57 (0.40–0.80)
Hypertension	268 (82.7%)	226 (57.4%)	3.56 (2.51–5.05)
Diabetes mellitus	148 (45.7%)	63 (16.0%)	4.42 (3.12–6.25)
Hyperlipidemia	152 (46.9%)	135 (34.3%)	1.70 (1.25–2.29)
WHR (female:> 0.8, male:> 1.0)	150 (46.3%)	144 (36.5%)	1.50 (1.11–2.02)
HDL-C < 1.0 mmol/L	86 (26.5%)	55 (14.0%)	2.23 (1.53–3.25)
Apo B/Apo A1 > 0.9	133 (41.0%)	47 (11.9%)	5.14 (3.53–7.49)
Hs-CRP ≥ 5.0 mg/L	277 (85.5%)	40 (10.2%)	52.16 (33.26–81.80)

*Abbreviation*: *MIPA* moderate-intensity physical activity, *WHR* waist-to-hip ratio, *HDL-C* high-density lipoprotein cholesterol, *Apo B* apolipoprotein B, *Apo A1* apolipoprotein A1, *Hs-CRP* high-sensitivity C-reactive protein

We analysed the association between all the modifiable risk factors and IS and tested the homogeneity in different ethnic groups with the Breslow-Day test. The results showed that only the difference in the association between a higher ApoB/ApoA1 ratio and first-ever IS in the Tujia and Miao populations was statistically significant (*P* < 0.05) (Table 3).

**Table 3:** The Homogeneity of the Association Between Risk Factors and Ischaemic Stroke by Ethnicity

Risk factor	Tujia			Miao			Han			*P*_*1*_	*P*_*2*_	*P*_*3*_
	Case (N = 178)	Control (*N* = 188)	Crude OR (95%CI)	Case (N = 95)	Control (*N* = 137)	Crude OR (95%CI)	Case (N = 51)	Control (*N* = 69)	Crude OR (95%CI)			
Manual Worker	144	158	0.80 (0.47–1.38)	76	127	0.32 (0.14–0.71)	37	57	0.56 (0.23–1.34)	0.482	0.351	0.058
Education ≥9 years	53	51	1.14 (0.72–1.79)	27	21	2.19 (1.15–4.18)	18	14	2.14 (0.94–4.87)	0.185	0.965	0.102
Given birth to 3 or more children	98	77	1.77 (1.17–2.67)	48	65	1.13 (0.67–1.91)	14	21	0.87 (0.39–1.93)	0.119	0.582	0.191
Monthly family income≥¥5000	80	39	3.12 (1.97–4.94)	42	14	6.96 (3.51–13.82)	26	18	2.95 (1.37–6.35)	0.901	0.101	0.055
Current smoking	84	61	1.86 (1.22–2.84)	48	61	1.27 (0.75–2.15)	23	33	0.90 (0.43–1.85)	0.088	0.443	0.269
Eating fast food frequency ≥ once per week	20	5	4.63 (1.70–12.63)	15	9	2.67 (1.12–6.38)	6	2	4.47 (0.86–23.13)	0.970	0.585	0.412
Eating hot Pot frequency ≥ once per week	24	16	1.68 (0.86–3.27)	12	6	3.16 (1.14–8.74)	4	2	2.85 (0.50–16.21)	0.573	0.921	0.306
MIPA	133	158	0.56 (0.34–0.94)	61	105	0.55 (0.31–0.97)	34	55	0.51 (0.22–1.16)	0.845	0.890	0.948
Hypertension	149	110	3.64 (2.23–5.96)	77	81	2.96 (1.60–5.48)	42	35	4.53 (1.92–10.72)	0.666	0.428	0.604
Diabetes mellitus	84	33	4.18 (2.60–6.77)	42	21	4.38 (2.36–8.11)	22	9	5.06 (2.07–12.36)	0.718	0.794	0.916
Hyperlipidemia	86	62	1.90 (1.25–2.90)	44	43	1.89 (1.10–3.24)	22	30	0.99 (0.48–2.05)	0.127	0.162	0.983
WHR (female:> 0.8, male:> 1.0)	87	82	1.24 (0.82–1.87)	41	43	1.66 (0.96–2.86)	22	19	2.00 (0.93–4.29)	0.279	0.700	0.396
HDL-C < 1.0 mmol/L	50	32	1.90 (1.15–3.14)	23	13	3.05 (1.46–6.38)	13	10	2.02 (0.81–5.06)	0.913	0.166	0.302
Apo B/Apo A1 > 0.9	79	17	8.03 (4.50–14.33)	35	22	3.05 (1.64–5.66)	19	8	4.53 (1.79–11.48)	0.304	0.487	0.024
Hs-CRP ≥ 5.0 mg/L	159	26	52.14 (27.75–97.97)	75	11	42.96 (19.51–94.58)	43	3	118.25 (29.71–470.68)	0.287	0.208	0.707

*P*_*1*_ value for Tujia vs Han, *P*_*2*_ value for Miao vs Han, *P*_*3*_ value for Tujia vs Miao

### Multivariate logistic regression analysis of risk factors for first-ever ischaemic stroke

Sex, age, ethnicity and all 15 risk factors that showed a significant association with IS in the univariate comparisons were included in the binary multivariate logistic regression analysis. In the entire population, six significant risk factors for first-ever IS included (in decreasing order of risk) a higher hs-CRP level, a higher monthly family income, an increased frequency of hot pot consumption, DM, a higher ApoB/ApoA1 ratio, and HT. Moderate-intensity physical activity was significantly associated with a reduced risk of IS (Table 4).

**Table 4:** Multivariate Logistic Regression Analysis of Risk Factors for First-Ever Ischaemic Stroke

Risk factor	*b*	*S*_*b*_	Wald χ^2^	Adjusted OR(95%CI)	*P*
Monthly family income≥¥5000	1.43	0.28	25.65	4.18 (2.40–7.28)	0.000
Eating hot Pot frequency ≥ once per week	1.06	0.45	5.71	2.90 (1.21–6.93)	0.017
MIPA	−0.70	0.30	5.48	0.50 (0.28–0.89)	0.019
Hypertension	0.93	0.28	10.69	2.52 (1.45–4.40)	0.001
Diabetes mellitus	0.96	0.29	10.99	2.62 (1.48–4.62)	0.001
Apo B/Apo A1 > 0.9	0.96	0.32	9.03	2.60 (1.39–4.85)	0.003
Hs-CRP ≥ 5.0 mg/L	3.92	0.27	210.63	50.54 (29.76–85.85)	0.000

*Abbreviation*: *MIPA* moderate-intensity physical activity, *Apo B* apolipoprotein B, *Apo A1* apolipoprotein A1, *Hs-CRP* high-sensitivity C-reactive protein. OR is adjusted for sex and age

### Additive interaction analysis between risk factors and ethnicity for first-ever ischaemic stroke

For the interaction analysis, let Tujia = 1 and Miao = 0, ApoB/ApoA1 > 0.9 = 1 and ApoB/ApoA1 ≤ 0.9 = 0, and OR_00_ = 1. In the R program, the function used a general linear model to estimate ORs with 95% CIs from a logistic regression model. Then, we used the epi.interaction function to calculate additive interactions by means of RERI, AP and SI with 95% CIs. The results of the interaction analysis showed that there was a biological interaction between the ApoB/ApoA1 ratio and ethnicity in the Tujia and Miao populations. The risk of IS related to the ApoB/ApoA1 ratio was significantly higher in the Tujia population (OR 8.91, 95% CI 4.84–16.39) than in the Miao population (3.05, 1.64–5.66) (Table 5).

**Table 5:** Additive interaction analysis between ApoB/ApoA1 ratio and ethnicity for first-ever ischaemic stroke

Ethnicity	OR(95%CI)		RERI(95%CI)	AP(95%CI)	SI(95%CI)
	ApoB/ApoA1 ≤ 0.9	ApoB/ApoA1 > 0.9			
Miao	1.00(Ref.)	3.05 (1.65–5.72)	5.75 (0.58 ~ 10.92)	0.65 (0.38 ~ 0.91)	3.66 (1.35 ~ 9.93)
Tujia	1.11 (0.75–1.66)	8.91 (4.94–16.82)			

*Abbreviation*: *Apo B* apolipoprotein B, *Apo A1* apolipoprotein A1, *RERI* the relative excess risk due to interaction, *AP* the attributable proportion due to interaction, *SI* the synergy index

## Discussion

In this case-control study, we found marked differences in IS risk profiles among the Han, Tujia, and Miao populations, which may help clinicians establish population-specific IS prevention programmes and aid in future research.

Ischaemic stroke is mainly caused by atherosclerosis and thrombotic obstruction of cerebral blood flow [25–28]; therefore, the identification of biomarkers for atherosclerosis-arterial stenosis or occlusion is essential for the early prevention of stroke. Considerable clinical observational studies of different populations have demonstrated that serum hs-CRP is a useful and powerful inflammatory marker in predicting future cardiovascular and cerebrovascular events [21, 29–33]. Our findings are in accordance with those of previous studies. A higher hs-CRP level was first demonstrated as an independent predictor for IS in all ethnic groups of the Xiangxi Tujia and Miao Autonomous Prefecture. Although CRP should be considered only a surrogate biomarker of upstream cytokines (IL-6 and IL-1β) [34], the classic acute-phase reactant that can be measured with high-sensitivity assays seems to be relevant for risk prediction. Specifically, higher hs-CRP levels appeared to increase the risk of recurrent stroke and vascular events in a hs-CRP sub-study in J-STARTS [35], and hs-CRP levels may be useful in individuals classified as “intermediate risk for IS by traditional risk factors” in the Atherosclerosis Risk in Communities (ARIC) study [36]. The ARIC study suggested that measurement of the plasma hs-CRP concentration could be used as an adjunct for risk assessment in primary and secondary prevention of IS, especially in different ethnic populations. However, in our study, we did not find that a higher hs-CRP concentration and ethnicity had a significant additive interaction effect on first-ever IS in the Han, Tujia and Miao populations.

Consistent with previous studies, our findings showed that HT and DM were significant risk factors for IS [5, 37]. HT and DM are global epidemics and have been recognized as common modifiable risk factors for both cardiovascular disease and stroke. Some studies have found marked ethnic differences in the association between BP parameters and stroke [38–40]. A 10-mmHg difference in systolic blood pressure (SBP) was associated with an 8% (95% CI, 0–16%) increase in stroke risk for Caucasian individuals but a 24% (95% CI, 14–35%) increase for African American individuals (P_interaction_ = 0.02) [40]. Limited data exist on ethnic differences in the association between HT and stroke in China, and in our study, there was no additive interaction effect on first-ever IS between HT and ethnicity in the Han, Tujia and Miao populations.

In the multivariate analyses in our study, the ApoB/ApoA1 ratio was a stronger risk predictor of IS than hyperlipidaemia, the WHR and HDL-C levels. An elevated ApoB/ApoA1 ratio mirrors an adverse imbalance between proatherogenic and antiatherogenic lipoprotein particles, which may result in enhanced atherosclerotic burden [41, 42]. There are still controversies regarding lipid profile results as risk markers of IS events [43–45], and recent evidence from various large studies suggested that the ApoB/ApoA1 ratio was better than the lipid profile in detecting IS risk [46, 47], which is in agreement with our results. A study demonstrated that an increased ApoB/ApoA1 ratio was independently associated with the occurrence of IS in young patients [48]. Furthermore, in our study, we found that the ApoB/ApoA1 ratio and ethnicity had an additive interaction effect on first-ever ischaemic stroke (RERI 5.75, 95% CI 0.58 ~ 10.92). The ethnic difference in the impact of the ApoB/ApoA1 ratio on stroke risk may be related to genetics or metabolism. Further studies are needed to clarify this mechanism. Overall, there is a clear need to focus on the ApoB/ApoA1 ratio to improve the prevention and treatment of IS in patients of different ethnicities.

In our study, we also found that higher monthly family income and increased frequency of hot pot consumption were significantly associated with IS. Hot pot is a local cuisine in the Tujia and Miao ethnic populations, and it appears on tables all year round in the Xiangxi Tujia and Miao Autonomous Prefecture. The broth of the Tujia and Miao-style hot pot is always chock-full of Sichuan peppercorns, chili pepper, and sour pickles. Moreover, most of the ingredients are salted or pickled foods, such as pickled fish, pork and chicken. Hot pot was first identified as an important risk factor for IS, and further research is needed to clarify the mechanism. A recent study demonstrated that moderate-to-vigorous physical activity doses equivalent to meeting the current recommendations attenuate or effectively eliminate the association between sitting and all-cause and cardiovascular disease (CVD) mortality risk among the least physically active adults [49], similar to the findings reported by the current study. This study suggested that targeted interventions that promote physical activity and a healthy diet for different ethnic populations could substantially reduce the burden of IS.

### Limitations and strengths

Our study has several potential limitations. First, after controlling for the possible confounding variables (age and sex) by multivariable logistic regression, there may be some potential confounding factors, such as exposure to indoor and outdoor pollution, stress at work and home, social support, and life events, that have not been taken into account. Second, our sample size may be inadequate to provide reliable information in subgroup analysis (in different ethnicities) and interaction analysis. Third, the OR from a case-control study is less precise than relative risk (RR) from a cohort study, so the use of OR in the additive interaction model was inferior to RR.

This study also has some strengths. Our study included the unique ethnic customs of Tujia and Miao populations as risk factors, such as local cuisine, which may add substantial information to other commonly modifiable risk factors.

## Conclusions

In conclusion, we provide the first evidence of ethnic variations among the Han, Tujia, and Miao populations in the effect of risk factors for IS in the Xiangxi Tujia and Miao Autonomous Prefecture. Therefore, we propose multidimensional IS prevention approaches among diverse populations, encompassing the population, community, health system, and individual levels. Racial and ethnic categories may help interpret health-related data, including surveillance and research, and are important in ensuring that IS prevention programmes remain generalizable to diverse populations.

## Supplementary Information

**Additional file 1.** Questionnaire for controls and patients with first-ever ischaemic stroke.

**Additional file 2: Table S1**: The name and value of variables in univariate analysis and multivariate logistic regression analysis.

## Data Availability

The datasets used and/or analysed during the current study are available from the corresponding author on reasonable request.

## Abbreviations

TOAST: Trial of Org 10,172 in Acute Stroke Treatment
NIHSS: National Institutes of Health Stroke Scale
CT: Computed tomography
MRI: Magnetic resonance imaging
ECG: Electrocardiography
MIPA: Moderate- intensity physical activity
WHR: Waist-to-hip ratio
HDL-C: High-density lipoprotein cholesterol
Apo B: Apolipoprotein B
Apo A1: Apolipoprotein A1
Hs-CRP: High-sensitivity C-reactive protein
RERI: The relative excess risk due to interaction
AP: The attributable proportion due to interaction
SI: The synergy index
