# Supplementary material for: Additive interaction between potentially modifiable risk factors and ethnicity among individuals in the Han, Tujia and Miao populations with first-ever ischaemic stroke
Source: BMC Public Health. 2021 Jun 3;21:1059. doi: 10.1186/s12889-021-11115-x (PMC8173719; doi:10.1186/s12889-021-11115-x)
Supplement: Supplementary file 1 — Additional file 1. Questionnaire for controls and patients with first-ever ischaemic stroke. [file 12889_2021_11115_MOESM1_ESM.docx]

Questionnaire for controls and patients with first-ever ischaemic stroke

The name of hospital:____________________________(only for patients)

Number:

Adimission Number: (only for patients)

**Please tick the corresponding option with “√” or fill in the corresponding words on the line.**

Patient’s Name:__________________ Telephone number:_____________

Proxy respondent’s Name:__________ Telephone number:____________

Family address:______________________________________________

I. Basic information

1. Birthdate(yyyy/mm/dd):___________________

2. Sex: ①male ②female

3. Ethnicity: ① Tujia ② Miao ③ Han

4. Occupation: ① mental worker ② manual worker

5. Education:

① <9 years of school education

② ≥9 years of school education(senior high school or above)

6. The number of fertility: ____________children

7. Monthly family income: ① <￥5000 ② ≥￥5000

II. Lifestyle

1. Smoking status:

① never smoked

② current smoking (smoking ≥1 cigarette per day within the year prior to the interview, including those who had quit smoking less than a year prior)

2. Eating fast food frequency ≥once per week: ① No ② Yes

3. Eating hot pot frequency ≥once per week: ① No ② Yes

4. Moderate-intensity physical activity: 4 hours or more per week, including brisk walking, dancing, gardening, housework and domestic chores, traditional hunting and gathering, general building tasks (e.g., roofing, thatching, painting), carrying/moving moderate loads (<20 kg), and so on.

① No ② Yes

III. History of diseases

1. Hypertension: under treatment with antihypertensive medication, a previous HT diagnosis, or current HT according to the 2003 WHO criteria (blood pressure of 140/90 mmHg or higher).

① No ② Yes

2. Diabetes mellitus: with a history of diabetes mellitus or treated diabetes preceding ischaemic stroke, diabetes was defined according to the 1999 WHO criteria as fasting plasma glucose (FPG) level ≥7.0 mmol/L (126 mg/dL), a 2-h oral glucose tolerance test of ≥11.1 mmol/L (200 mg/dL), or glycated haemoglobin (HbA1c) level ≥ 6.5%.

① No ② Yes

3. Hyperlipidemia: total cholesterol (TC) level ≥6.2 mmol/L (240 mg/dL), triglyceride (TG) level ≥2.3 mmol/L (200 mg/dL), low-density lipoprotein cholesterol (LDL-C) level ≥4.1 mmol/L (160 mg/dL), or high-density lipoprotein cholesterol (HDL-C) level <1.0 mmol/L (40 mg/dL).

① No ② Yes
