# Supplementary material for: Additive interaction between potentially modifiable risk factors and ethnicity among individuals in the Han, Tujia and Miao populations with first-ever ischaemic stroke
Source: BMC Public Health. 2021 Jun 3;21:1059. doi: 10.1186/s12889-021-11115-x (PMC8173719; doi:10.1186/s12889-021-11115-x)
Supplement: Supplementary file 2 — Additional file 2: Table S1: The name and value of variables in univariate analysis and multivariate logistic regression analysis. [file 12889_2021_11115_MOESM2_ESM.docx]

Additional file 1

Table S1 The name and value of variables in univariate analysis and multivariate logistic regression analysis

| Variable name | Value |
| --- | --- |
| Y | 1=case, 0=control |
| Ethnicity | 1=Tujia, 2=Miao, 3=Han; reference group=3 |
| Occupation | 1=manual worker, 0=mental worker |
| Education≥9 years | 1=Yes, 0=No |
| The fertility number | 1=given birth to 3 or more children  0=given birth to 2 or less children |
| Monthly family income | 1=≥￥5000, 0=<￥5000 |
| Smoking | 1= Current smoking, 0=Never smoked |
| Eating fast food frequency ≥ once per week | 1=Yes, 0= No |
| Eating hot Pot frequency ≥ once per week | 1=Yes, 0= No |
| Moderate-intensity physical activity(MIPA) | 1=Yes, 0=No |
| Hypertension | 1=Yes, 0=No |
| Diabetes mellitus | 1=Yes, 0=No |
| Hyperlipidemia | 1=Yes, 0=No |
| Waist-to-Hip ratio | male：1=>1.0, 0=≤1.0  female：1=>0.8, 0=≤0.8 |
| High-density lipoprotein cholesterol(HDL-C) | 1=<1.0mmol/L, 0=≥1.0mmol/L |
| Apolipoprotein(Apo)B/ApoA1 | 1=>0.9, 0=≤0.9 |
| High-sensitivity C-reactive Protein (hs-CRP) | 1=≥5.0mg/L, 0=<5.0mg/L |
